# The Role of Hip Joint Clearance Discrepancy as Other Clinical Predictor of Reinjury and Injury Severity in Hamstring Tears in Elite Athletes

**DOI:** 10.3390/jcm10051050

**Published:** 2021-03-04

**Authors:** Jesus Seco-Calvo, Martin Palavicini, Vicente Rodríguez-Pérez, Sergio Sánchez-Herráez, Luis Carlos Abecia-Inchaurregui, Juan Mielgo-Ayuso

**Affiliations:** 1 Institute of Biomedicine (IBIOMED), Physiotherapy Department, University of Leon, Campus de Vegazana s/n, 24071 Leon, Spain; luiscarlosabecia@gmail.com; 2 Departament of Kinesiology, College of Health and Human Services, California State University, 2345 E, San Ramon M/S MH26, Fresno, CA 93740, USA; coachpalavicini@yahoo.com; 3 Physiotherapy Department, University of Salamanca, Campus Miguel de Unamuno, c/Donantes de Sangre s/n, 37007 Salamanca, Spain; vicente.rodriguez@usal.es; 4 Servicio de Cirugía y Traumatología Ortopédica, Complejo Asistencial Universitario de León (CAULE), Altos de Nava s/n, 24071 León, Spain; herraezsergios@yahoo.es; 5 Department of Health Sciences, Faculty of Health Sciences, University of Burgos, 09001 Burgos, Spain; jfmielgo@ub.es

**Keywords:** risk factors, hamstring tear, head femoral height discrepancies

## Abstract

Hamstring tear injuries (HTI) are the most prevalent injuries in athletes, with high reinjury rates. To prevent reinjury and reduce the severity of injuries, it is essential to identify potential risk factors. Hip characteristics are fundamental to optimal hamstring function. We sought to investigate the role of hip joint clearance discrepancy (JCD) as a risk factor for HTI and a clinical predictor of risk of reinjury and injury severity. A cross-sectional, retrospective study was performed with elite athletes (*n* = 100) who did (*n* = 50) and did not (*n* = 50) have a history of injury. X-rays were taken to assess JCD. We reviewed muscular lesions historial, and health records for the previous 5 years. Significant differences were found in injury severity (*p* = 0.026; ŋ^2^*p* = 0.105) and a number of injuries (*p* = 0.003; ŋ^2^*p* = 0.172). The multivariate analysis data indicated that JCD was significantly associated with the number of injuries and their severity (*p* < 0.05). In the stepwise regression model, JCD variability explained 60.1% of the number of injuries (R^2^ 0.601) and 10.5% of injury severity (R^2^ 0.0105). These results suggest that JCD could play an important role as a risk factor for HTI and also as a clinical predictor of reinjury and injury severity.

## 1. Introduction

The epidemiology of hamstring muscle injury is well known [1,2,3]: Injuries are much more common in the biceps femoris (BF) than the medial hamstring [4], in particular, affecting the biceps femoris long head (BFlh) [5], and there are no significant sex differences [6]. In this context, a hamstring tear injury (HTI) is the most common reason for track athletes taking time out from training and competition [7,8] and various factors have been suggested to explain the high rate of reinjury of this muscle [9]. 

Numerous factors may predispose athletes to HTI [3,10], and there is consensus that the causes are multifactorial [11], and likely interrelated [12], with certain anatomical and architectural features playing a role [13]. Nonetheless, the risk factors most consistently associated with HTI are [14,15,16] older age, a history of hamstring injury, and higher quadriceps peak torque, short BFlh fascicles, and eccentric knee flexor weakness being the key factors that increase the risk of recurrent HTI [17]. Moreover, certain risk factors (decreased quadriceps flexibility and time to walk pain-free, as well as a history of hamstring injury and older age) have been identified as predictors of clinical outcome [15,16,18,19]. Given this, it is important to explore athletes’ history of injuries, as well as their anthropometric and physical characteristics [20].

Programs for preventing this type of injury have shown to be effective in reducing rates of HTI in athletes [21,22,23,24,25,26,27], but data concerning which HTI risk factors should be used for selecting athletes for such programs are limited. Specifically, some studies have not considered confounders [11,14], e.g., morphological, and architectural characteristics [13,20]. Furthermore, while some clinically useful diagnostic tests have been suggested [28,29,30], few studies have examined the association between HTI and hip range of movement [14,15,16,19,31] or pelvic parameters [31,32]. However, recent studies [33,34,35,36,37] have shown that the characteristics of the hip are critical for optimal hamstring function [38]. In this sense, it has been reported [39] that the hamstring muscles can restrict hip flexion, especially when the knees are extended. It was hypothesized that people with short or tight hamstrings would have an abnormal pelvic tilt in some hip flexion postures [39,40]. Furthermore, it has been suggested that some conditions, such as ischiofemoral impingement, present symptoms similar to those of HTI [41,42], so this pathophysiological relationship should be considered. Thus, an evaluation of pelvic parameters [43] can help identify morphotypes with increased risk of injury, but to our knowledge, the influence of JCD on this type of injury has yet to be investigated.

In addition, it has been shown that to achieve optimal sprinting performance, athletes use mechanisms with eccentric and concentric muscle actions to take advantage of the elastic component of the muscle action and to improve the production of muscle strength around the hip [43,44,45]. Thus, at the beginning of the sprint, a stretch-shortening cycle is executed centered on the hip extensor muscles [45]. In this sense, it has also been shown that the center of mass is relevant to be able to develop the necessary power to obtain acceleration while running [46]. However, the role played by the muscle strength to be performed and the morphological variability of the femoroacetabular joint, such as joint clearance discrepancy (JCD), as well as the relationship between technique and performance remain largely unexplored [47]. Furthermore, Handsfield et al. (2017) [48] and Brazil et al. (2018) [49] indicated that physical abilities and anatomical factors are often overlooked in sprinting kinetic analyzes.

Given the need to strengthen prevention of this common injury, a new approach is required and for this reason, the objective of our study was to explore the potential role of JCD as a risk factor for HTI and as a clinical predictor of reinjury and injury severity. Therefore, the hypothesis of this study was that the presence of JCD could be related to a higher frequency of injury of HTI.

## 2. Experimental Section

### 2.1. Study Design and Participants

We conducted a cross-sectional study and reported it here in accordance with the Strengthening the Reporting of Observational Studies in Epidemiology (STROBE) statement. The study was assessed with reference to the consensus statement of epidemiological studies in athletics [50].

The participants were 100 elite US athletes (40% women, 60% men) who underwent an X-ray of the pelvis to assess the presence of JCD. They were divided into two groups based on whether they had a history of HTI: Those who had had HTI in group 1 (G1) (*n* = 50; age: 26.4 ± 7.1 years; male, *n* = 35 [70.0%]; female, *n* = 15 [30.0%]) and those who had not in group 2 (G2) (*n*^2^ = 50; age: 26.3 ± 6.5 years; men, *n* = 25 [50.0%]; women, *n* = 25 [50.0%]).

### 2.2. Procedure

Participants’ history of HTI over the previous 5 years was assessed by clinical interview and review of their medical records. In accordance with the Munich consensus statement [51], we use the term ‘tear’ to describe the hamstring injuries. The injuries were graded following the new system proposed by the British Athletics Muscle Injury Classification [52]. For our analysis, participants were stratified by grade of injury, grouped “small-to-moderate” (Grades 1 and 2) and “extensive-to-complete” (Grades 3 and 4), as well as by sex, race, event type (track distance, and with or without hurdles), and the number of times they had had this type of injury (<2, 2, >2). Lower limb length was measured on X-ray [38].

**Figure 1:**
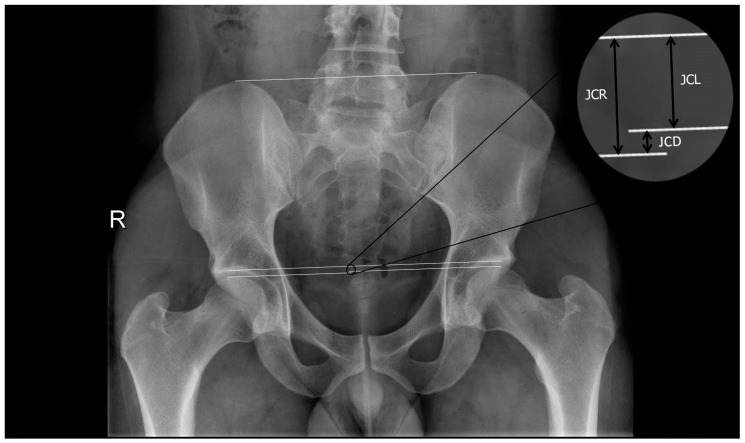
X-rays to quantify difference in the joint clearance. JCD: Joint clearance discrepancy; JCL: Joint clearance left; JCR: Joint clearance right.

X-rays were performed in an anterior-posterior projection and the axial projection proposed by Johnson to determine JCD. Concretely, it was quantified the difference between the femoral head height and the acetabulum by the distance between the upper limit of the femoral head and the lower limit of the cup, measurement taken in the anterior part of the cup (Figure 1). Measurements were made using a Gonstead Spinograph Parallel Ruler^®^ (Wellness Operation Company LLC, Boynton Beach, FL, USA).

### 2.3. Inclusion Criteria

Participants were US athletes who had qualified for the US National Championships. The US National Championships qualifying performances are at least equal to the current World Championships qualifying standards. Juniors (athletes under 20 years of age) had competed in US Junior Nationals and achieved the minimum score necessary for them to complete in their category at the World U20 Championships. For Olympic years, the scores for classifying for national championships were considered Olympic trials. Given this, the definition of “elite” in our study was given to athletes who met the US National Championships/World Championships qualifying standards (see Supplementary).

In addition, participants were required to meet the following criteria: Values within the normal range for the acetabular index, also called acetabular roof angle or Tönnis angle, values >3° and <13° being considered normal [53]; horizontal toit externe angle (i.e., the orientation of the acetabular roof and the coverage of the femoral head), values <10° and >0° being considered normal; and alpha angle (α), <50° being considered normal; negative Drehmann sign and impingement tests; and no history of hip surgery or trauma.

### 2.4. Exclusion Criteria

Athletes were excluded if they had current or past history of femoroacetabular impingement (FAI), Perthes disease, bone dysplasia, scoliosis, acetabular retroversion, coxa profunda, protrusio acetabuli, os acetabuli, or chronic hip dysplasia, or any radiographic signs suggestive of FAI [54].

### 2.5. Statistical Procedure

Statistical analysis was performed using IBM SPSS (version 24.0, IBM Corporation, Armonk, NY, USA). Results are reported as mean and standard deviation. Values of *p* < 0.05 were considered significant.

Kolmogorov–Smirnov tests were carried out to test the normality of the data. Differences in sociodemographic, characteristics, and the type of athletic event between G1 and G2 were assessed with the chi-square test.

To analyze whether JCD was associated with number of injuries or injury severity, a stepwise regression analysis was used with JCD as the independent variable and number of injuries and injury severity outcomes as the dependent variables.

Differences between G1 and G2 in age and anthropometric characteristics were assessed by one-factor (univariate) analysis of variance. Similarly, differences in JCD among athletes with a history of HTI were assessed by one-factor (univariate) analysis of variance with different descriptive and athletic variables as the fixed factor. A Bonferroni post-hoc test was performed for pairwise comparisons between groups. Differences between athletes ≤ 2 injuries and athletes with > 2 injuries, were performed using one factor univariant ANOVA test. Partial square eta (η^2^*p*) was used as a measure of effect size and interpreted as: (I) no effect: 0 ≤ η^2^*p* < 0.05; (II) minimum effect: 0.05 ≤ η^2^*p* < 0.26; (III) moderate effect: 0.26 ≤ η^2^*p* < 0.64; and (IV) strong effect: η^2^*p* ≥ 0.64 [55].

Bivariate correlations of JCD with the number of injuries, injury severity, and with lower limb length were tested using Pearson’s product-moment correlation, and regression lines and 95% confidence intervals were also calculated. 

### 2.6. Ethical Considerations and Participant Involvement 

Although, according to Spanish Law, (Act 14/2007 of 3 July on biomedical research, Order SAS/3470/2009, of 16 December, official state gazette 310, of 25 December [Royal Legislative Decree 2009, 2577]) this study did not need to be approved by an Institutional Review Board because it was an observational study that did not require any changes to standard clinical practice, and data that were analyzed for the study did not contain any personal data which might reveal patient identity, the study was approved by the Ethics Committee of the University of León (Spain) (identification code ETICA-ULE-026-2020). 

All procedures were carried out in accordance with the Declaration of Helsinki (2013, revised 5 May 2015), ethical regulations and Spanish law on the protection of personal data (15/1999) and biomedical research in humans (14/2007). We explained the procedure and objectives of the study to the athletes and all participants provided written informed consent to X-ray examinations and the use of demographic and clinical data related to their care for the purposes of this study. 

### 2.7. Patient and Public Involvement

Patients were not, however, invited to comment on the study design, consulted to develop patient-relevant outcomes, or interpret the results, or invited to contribute to the editing of this paper.

## 3. Results

### 3.1. Characteristics of the Study Sample and between Group Differences

The characteristics of the athletes included are summarized in Table 1 and Table 2 by group, i.e., whether athletes had a history of HTI. Notably, our data show that the only significant difference in anthropometric characteristics between the groups with and without a history of injury was the presence of JCD (*p* < 0.001; ŋ^2^*p* = 0.549) (Table 1). Specifically, athletes with a history of HTI had a significantly larger JCD (0.88 ± 0.40 cm vs. 0.18 ± 0.47 cm in athletes with no history of HTI). The only significant difference in sociodemographic characteristics and athletic event type found was in sex distribution (*p* < 0.041). 

Table 3 reports JCD among athletes with a history of HTI stratified by sociodemographic, anthropometric, and athletic data. In this analysis, it can observe that differences reached significance for race (*p* = 0.017), injury severity (*p* = 0.026), and number of injuries (*p* = 0.003). Specifically, among athletes with a history of HTI, JCD was significantly larger in black than in white athletes, and those who had had serious injuries (vs minor injuries) and/or >2 injuries (vs ≤2 injuries). 

### 3.2. Relationship of History of Injury with JCD

The multivariate analysis (Table 4) indicated that JCD was significantly associated with the number of injuries and their severity (*p* < 0.05). In the stepwise regression model, JCD variability explained 60.1% of the number of injuries and 10.5% of injury severity. 

**Figure 2:**
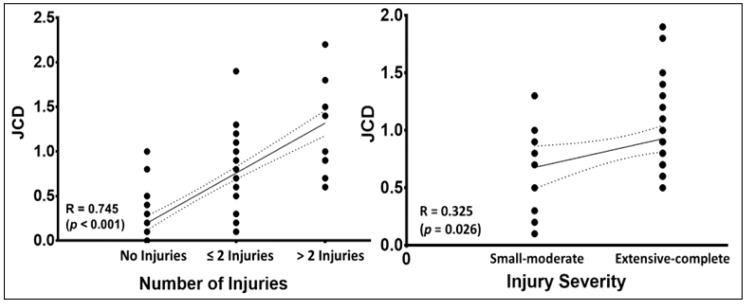
Pearson’s correlation (±95% confidence intervals) between joint clearance discrepancy (JCD) and number of injuries (**left**) and injury severity (**right**).

Figure 2 shows a strong positive correlation between JCD and number of injuries (R = 0.745; *p* < 0.001). Furthermore, there was a moderate positive correlation between JCD and injury severity (R = 0.325; *p* = 0.026).

Table 5 shows that the athletes who presented more than two injuries had a JCD and a lower limb length significantly greater than the athletes who presented less than 2 injuries (*p* < 0.005 in both cases).

**Figure 3:**
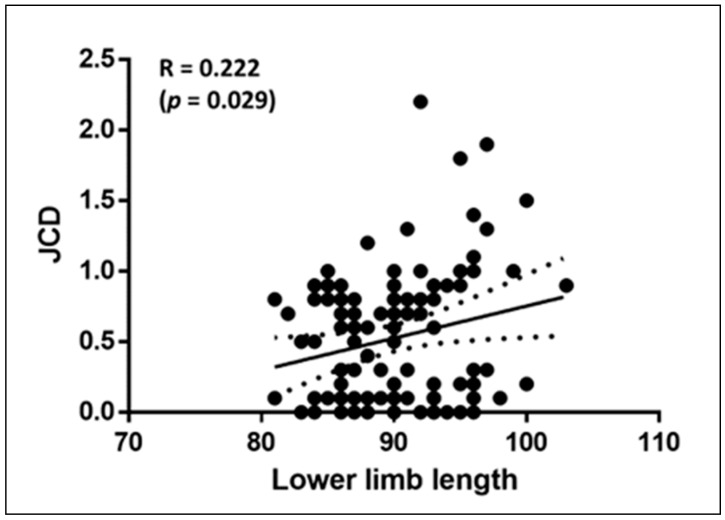
Pearson’s correlation (±95% confidence intervals) between joint clearance discrepancy (JCD) and lower limb length.

Figure 3 displays a significant positive Pearson’s correlation between JCD and lower limb length (R = 0.222; *p* = 0.029).

Table 6 indicates that athletes with serious injury were of a significantly younger age (*p* = 0.040), as well as a higher JCD (*p* = 0.026) than athletes with slight injury.

## 4. Discussion

To the best of our knowledge, this study has found the first evidence for a role of JCD as a risk factor for HTI and clinical predictor of reinjury and injury severity. In fact, the multivariate analysis completed, indicated that JCD was significantly associated with the number of injuries and their severity. Concretely, 60.1% of the number of injuries and 10.5% of injury severity was explained by JCD variability. In addition, a strong positive correlation between JCD and number of injuries were observed; furthermore, there was a moderate positive correlation between JCD and injury severity.

In recent studies [33,34,35,36,37], the hip has been shown to play a major role in optimal hamstring functioning, and an assessment of commonly used pelvic parameters such as pelvic incidence, pelvic tilt, sacral slope, pelvic obliquity, axial rotation of the pelvis, right femur torsion, left femur torsion, and leg length discrepancy, may help to identify morphological types at greater risk of this type of injury [38]. Nonetheless, previously, JCD has not been considered.

### 4.1. The Role of the Hip

For optimal hamstring functioning, it is clear that proper hip biomechanics are essential [34,56], lumbar-pelvic stability being considered an important factor in clinical practice and a modifiable risk factor for HTI. Nonetheless, according to a recent review, there is a paucity of evidence in this field [23]. It has been suggested that the reduced activation of the gluteus maximus and weakness of this muscle are risk factors for HTI [16,27]. On the other hand, Chumanov et al., (2007) [57] proposed a theoretical model for aberrant pelvic motion in hamstring injury which indicates that small increases in hip flexor activation, beyond that typically observed in sprint and hurdle races, increase the stretch in the BF and other hamstring muscles of the other leg in the late swing phase. These activities require the hamstring to contract or lengthen while in hip flexion and may result in provocative tensile and compressive load at the BF insertion [41]. In the case of the acceleration, take-off, landing, and deceleration movements required for hurdling, some athletes are able to keep their trunk vertical while negotiating the obstacles and others seem to absorb the forces at the hip and run comfortably, maintaining a wide range of motion, but a considerable number experience pain referred to the groin [58,59].

In this sense, it is known that the hip achieves great mobility and stability during various activities and that its participation in specific gestures requires a complex range of hip movements and muscle activity [60]. Thus, it has been reported that a greater amount of internal/external rotation of the hip occurs during torsion [60], and that physiological bilateral torsion requires a wide range of axial rotation of the hip, producing the greatest part of the joint range of hip flexion [61], as occurs in hurdlers. In this sense, according to the results obtained in this study, the presence of JCD could limit the hip from achieving that great mobility and stability necessary, such as the changes of anterior/posterior pelvic tilt, causing the athlete to compensate with the activation of the major muscle, which could lead to HTI.

Although more studies are still required to confirm this, it has been suggested that anatomic variations in the femoroacetabular joint could cause pain referred to the hip or in the proximal insertion of the hamstrings and develop compensations in the kinematics of running [62]. In this context, it has been reported that the pattern of neuromuscular coordination (muscle activations patterns) of gait varies according to walking at slow speeds [63], so it could be suggesting that these patterns would also change at running speeds. Therefore, one might think that in the participants of the present study, with JCD, these modifications in the pattern of neuromuscular coordination and the development of this pathomechanical pattern could be the cause of HTI. In this perspective, many athletes have a somewhat reduced range of motion for hip flexion or internal rotation, these being common findings in FAI [64,65,66,67]. We should underline, however, that we excluded athletes with FAI or signs thereof [53,54], and despite this, our findings suggest a relation between JCD and HTI. 

### 4.2. Relationship of Hip Flexion with the Hamstring Muscle

Reduced hip flexion matters because the angular range of hip flexion is considered a key determinant of sprint and hurdle performance [68] and the role of hamstrings in achieving high hip flexion angles has been shown to be crucial [35]. Notably, the type of event and stretching to which hamstrings are subjected have a marked impact on treatment and prognosis in HTI [69]. Specifically, a potential complication of reduced hip flexion during sprinting is a compensatory increase in pelvic tilt which may, in turn, increase hamstring strain [70]. In sprinters and hurdlers, it is key to restoring normal patterns of movement. In relation to this, good motor control is essential and movement control training may be useful for the prevention of HTI [71,72]. It is known that progressive running drills overload the hamstrings functionally, gradually increasing the speed of movement and lengthening of the muscle [73]. In this context, in HTI, abnormalities have been observed in hip and pelvis movement, including reduced hip flexion [74] and increased anterior pelvic tilt [75].

As well as a good range of hip flexion [68], the ability to apply horizontal force [76] is key in sprint racing and it is for these reasons that short BFlh fascicles and eccentric knee flexor weakness are associated with an elevated risk of hamstring reinjury [17]. In line with this, Higashinara et al., (2019) [33] showed significant reductions in BFlh activation and muscle and tendon length in a previously injured leg during the late swing phase of sprinting, which may explain why short hamstrings are associated with high femoral head height.

### 4.3. The Influence of Training

As highly specific resistance training is important for the effective transfer of strength to sprinting performance [77], sprint training with added resistance is common practice. In this sense, ballast training could modify the athlete’s center of mass, which can be a great handicap to the rotation capacity of the hips due to an increase in their moment of inertia. This modification in the kinematics of sprinting, could affect not only performance but also the potential risk of injury [77]. Consequently, in athletes with JCD it could potentially be an added overload when sprinting, which could explain the greater injury severity in these athletes as in those of the present study. Therefore, when there is a great strain on the hamstrings, the incidence of injuries in sprinters may be related to the strain of the muscle-tendon unit [78]. Therefore, it is relevant to consider any potential effect of this type of training on hamstring strain.

### 4.4. Running Biomechanics

Optimal sprinting performance depends on reaching maximum horizontal power from the starting blocks (during block clearance) and increasing speed from that position [43]. On the other hand, the main period of energy generation of the hip extensors is in the initial posture [43]. Thus, during sprinting, athletes use a mechanism with eccentric and concentric muscular actions to take advantage of the elastic component of the muscular action and to improve the production of muscular force around the hip, knee, and ankle [43].

In this sense, in the starting position, sprinters show a higher average of force production during the push against the studs (blocks), especially from the rear leg and particularly from the hip, which seems to be important for performance [47]. Furthermore, it has been shown that the hip extensors of the supporting leg (biceps femoris) contribute, although only during the second pose, to force production [44]. Furthermore, the role of the sprinter’s body structure in the touchdown remains unclear, and the role of strength and anatomy in these associations between technique and performance also remains largely unexplored [47]. The athlete’s anthropometry is particularly important for the sprinter to present an optimal starting position. Thus, after the departure of the rear block, the front leg should also assist vertical movement, but its main function appears to be forward propulsion [47]. Next, both the hip and ankle reach maximum extension after takeoff [43]. In this sense, if the athlete presented a JCD, he could perform some type of aberrant compensatory movement, to maintain his acceleration and center of mass, placing a greater tension on the muscle-tendon unit of the hamstring. Thus, several authors [48,49] indicate that physical capabilities and anatomical factors are often overlooked in joint kinetic analyzes of sprinting. Therefore, taking into account the results of this study, anatomical assessments, such as the presence of JCD, should be incorporated into the athletes’ examinations in order to determine the risk of injury.

On the other hand, performance during the sprint acceleration phase depends on the anteroposterior net force generated during contact with the ground, which directly influences the anteroposterior acceleration of the center of mass. Furthermore, extraordinarily large increases in stride speed (step) occur during initial acceleration, due to the increase in the length of the stride, and subsequently due to the increase in the frequency of the step [46]. Therefore, as sprinters obtain a higher running performance, the shorter the contact time with the ground. To do this they must achieve a higher stride rate than is achieved with an upright torso and high knee lift, which allows sprinters to accelerate the foot down and back before touchdown (contact). This running mode requires a wide range of axial rotation of the hip and greater excitation of the muscle-tendon unit and considering that the presence of JCD could alter that harmony in the biomechanics of running, it could cause overexcitation in the running muscle, specifically the tendon unit of the hamstring and a muscle strain may occur [41].

### 4.5. The Age Factor

Although it is known that age is a consistent factor in terms of the risk of injury to HTI [17], this aspect must be qualified in this population. Debaere et al. (2017) [45] have shown that there is a significant difference between young and adult well-trained sprinters in specific technical skills. Young sprinters have more relative joint power offsets than the hip joint. On the other hand, young athletes may experience longer periods and with greater force of stretching of the rectus femoris due to a greater contribution of the hip joint in generating energy [45]. This could cause a greater increase in their potential for energy storage and tension in the muscle, the tendon unit of the antagonist muscles, that is the hamstring, and if the stretching capacity of the muscle is exceeded, it could cause injury. In addition, it has been reported that sprinters show a greater change in posture, with the horizontal center of mass during the first stance, which correlates with a longer stretch time and length of the muscle-tendon unit of the rectus femoris [79]. In this sense, these athletes (young sprinters) show greater technical ability to apply greater relative portions of the resulting external force in a horizontal direction, to control the center of mass [79], which could be compromised if the joint interline is reduced, which could explain why, in the present study, these athletes (young sprinters) with a JCD show a higher incidence of injury.

### 4.6. Muscular Mechanics

It remains a matter of debate which mechanical parameter best explains injuries due to muscle tension [56,80]. Animal studies [81] suggest that it is not a greater force per se which causes muscle damage after eccentric contraction but rather the magnitude of active tension (i.e., the tension during active lengthening) described in terms of the interaction between the myofiber cytoskeleton, sarcomere, and sarcolemma [81]. In line with this, some studies [82,83] found that a muscle subjected to repeated stretch-shortening cycles of constant muscle-tendon unit excursion had significantly different joint torque and fibre strain when the timing of activation or starting muscle length were changed. Hence, while muscle activation may be an important determinant of training-induced hypertrophy, the mode of contraction [84] seems to be a stronger driver of architectural changes in hamstrings [85], and these architectural changes produce muscle stiffness [58] and an active range of knee movement deficiency [86].

In relation to this, muscle contraction mechanics [87], and the isokinetic strength [83] may be modified by scar tissue that often forms along the musculotendinous junction at the site of past injuries [83,87]. In particular, collagen fibres in remodeled tendon tend to be less well organized than a normal tendon and have different stiffness properties [88]. Specifically, scar tissue may increase the overall mechanical stiffness of the tissue it replaces, meaning that compared to the pre-injury state, muscle fibres need to lengthen more to achieve the same overall musculotendon length [88]. In this sense, Fouasson-Chailloux et al. (2019) [83] have shown that after injury, a deficit of eccentric strength persists in time. It is therefore plausible that the risk of re-injury is increased by scar tissue at the site of past musculotendon injuries adversely affecting local tissue mechanics. This might explain why in, our study, reinjury was related to short hamstrings and was associated with a JCD.

In line with this, the muscle shortening-stretching mechanism during the first strides in sprinters can be variable due to the specific positional changes of each athlete. Furthermore, although joint energy generation and absorption is only indirectly related to each specific muscle action, these muscle shortening-stretching mechanisms indicate that during the first steps of the sprint, a stretching-shortening mechanism is centered on the hip and the ankle, but not the knee [43]. At the hip, the maximum power generation of the hip extensors is immediately before and at the start of contact, where the hip extensors actively pull the body over the point of contact. For this to be possible, the femoroacetabular joint clearance must be similar (similar) in both hips. If not, these stretch-shortening mechanisms may not be adequate, and the muscle may then undergo a sudden stretch, resulting in injury. This could explain why athletes with a JCD would be more susceptible to presenting a higher incidence of injury of HTI. 

### 4.7. Theory on the Pathophysiological Correlation about HTI and JCD 

Although the present study was a correlational study and did not permit us to determine cause-effect relationships, authors suggest some possible reasons to explain the provided positive association between JCD and HTI incidence. HTI are characterized by a mechanism of extreme hip flexion combined with knee extension [41]. Biomechanically, shorter hamstrings may produce a posterior tilt of the iliac bone increasing the JCD in the anterior region of the hip [40]. Pathologically, the ischiofemoral impingement [41,42], was considered as an extra-articular hip impingement syndrome accompanied by compression between the lesser trochanter and the ischial tuberosity in conjunction with a possible JCD increase secondary to synovial displacement and increase in the anterior part of the hip joint [89]. In addition, this synovial displacement could be favored in subjects with intrinsic risk factors to develop THI such as, age, body mass index, or genetic polymorphisms (e.g., COL5A1 that encodes for collagen type V) [41,42]. More studies are needed to provide more information about it and clarify this question.

## 5. Study Limitations

This study has some imitations. Importantly, rates of reinjury are high in athletes, often due to inadequate rehabilitation or premature return to competition, despite it being important for elite athletes, above all sprinters and hurdlers, to allow time for complete functional recovery before starting to compete [69], and we lack data to properly investigate the influence of these factors. Another limitation is the relatively small sample size, which hindered comparisons between subgroups of athletes who compete in different events.

Prospective studies are now needed to confirm whether the presence of JCD is a predictor of HTI or a result of athletes’ previous injuries. 

## 6. Implications for Practice 

The study results provide useful predictive information for clinicians involved in the diagnosis of hamstring tear injuries or responsible for the clinical assessment of athletes. In routine sports medicine check-ups and clinical examinations in athletes, an X-ray of the pelvis could be added allowing measurement of the joint clearance discrepancy, allowing athletes with this specific risk factor to be included in hamstring injury prevention programs. 

Perhaps this information could be of interest to scientific societies, such as ISMuLT (the “Italian Society of Muscles, Ligaments and Tendons”) and could be considered useful in the Management Guides for muscle injuries [90,91].

## 7. Conclusions

In conclusion, the results of the present study suggest that JCD could play a relevant role as a risk factor for HTI and also as a clinical predictor of risk of reinjury and injury severity. These findings may help identify which type of athlete is most likely to experience a hamstring tear.

## Figures and Tables

**Table 1:** Athletes’ age and anthropometric characteristics by group, i.e., those with and without a history of injury.

Variable	Injury (*n* = 50)	No Injury (*n* = 50)	*p*	ŋ^2^*p*
Age (years)	26.4 ± 7.1	26.3 ± 6.5	0.930	0.001
Height (m)	1.74 ± 0.09	1.74 ± 1.0	0.787	0.001
Body mass (kg)	63.5 ± 10.7	61.8 ± 11.7	0.444	0.006
Body mass index (kg/m^2^)	20.9 ± 2.6	20.4 ± 2.6	0.332	0.010
Upper body length (cm)	83.5 ± 5.3	82.8 ± 5.6	0.508	0.004
Lower body length (cm)	90.5 ± 5.5	90.7 ± 0.5	0.855	<0.001
Joint clearance discrepancy (cm)	0.88 ± 0.40	0.18 ± 0.47	<0.001	0.549

Results are presented as mean and standard deviation. *p*: *p*-value from one-way analysis of variance.

**Table 2:** Athletes’ sociodemographic characteristics and event type by group, i.e., those with and without a history of injury.

Variable		Injury (*n* = 50)	No Injury (*n* = 50)	*p*
Sex	Male	35 (70.0%)	25 (50%)	0.041
	Female	15 (30.0%)	25 (50%)	
Race	White	40 (80.0%)	36 (72.0%)	0.603
	Black	8 (16.0%)	12 (24.0%)	
	Asian	2 (4.0%)	2 (4.0%)	
Track distance	100–200 m	19 (38.0%)	17 (34.0%)	0.826
	>200 and ≤1500 m	18 (36.0%)	21 (42.0%)	
	>1500 m	13 (26.0%)	12 (24.0%)	
Hurdling	No	41 (83.7%)	38 (76.0%)	0.342
	Yes	9 (16.3%)	12 (24.0%)	

Results are presented as *n* and percentage. *p*: *p*-value from chi-square test.

**Table 3:** Joint clearance discrepancy (JCD) among athletes with a history of injury stratified by sociodemographic characteristics, event type, and nature of the injury.

		JCD	*p*	ŋ^2^*p*
Sex	Males (*n* = 35)	0.93 ± 0.45	0.177	0.038
	Females (*n* = 15)	0.76 ± 0.16		
Race	Caucasian (*n* = 40)	0.82 ± 0.33	0.017	0.158
	Black (*n* = 8)	1.22 ± 0.55 *		
	Asian (*n* = 2)	0.88 ± 0.40		
Track distance	100–200 m (*n* = 19)	0.86 ± 0.33	0.095	0.095
	>200 and ≤1500 m (*n* = 18)	1.02 ± 0.48		
	>1500 m (*n* = 13)	0.71 ± 0.30		
Hurdling	No (*n* = 41)	0.87 ± 0.41	0.703	0.003
	Yes (*n* = 9)	0.93 ± 0.32		
Injury severity	Small-moderate (*n* = 13)	0.68 ± 0.33	0.026	0.105
	Extensive-complete (*n* = 34)	0.93 ± 0.33		
Number of Injuries	≤2 injuries (*n* = 41)	0.80 ± 0.32	0.003	0.172
	>2 injuries (*n* = 9)	1.22 ± 0.53		
Leg injured	Right (*n* = 8)	0.85 ± 0.48	0.842	0.001
	Left (*n* = 42)	0.88 ± 0.39		

Results are indicated as mean and standard deviation. *p*: *p*-value from one-way analysis of variance. * Significant differences with respect to white after Bonferroni’s correction.

**Table 4:** Multivariate analysis with number of injuries and injury severity as the dependent variable and joint clearance discrepancy (JCD) as the predictor.

Model	Unstandardized Coefficients		Standardized Coefficients	*t* Value	*p* Value	95% Confidence Interval		R^2^
	B	SE	ß			Lower Limit	Upper Limit	
Number of Injuries								
(Constant)	0.092	0.115		0.795	0.429	−0.137	0.320	0.601
JCD	1.945	0.164	0.770	11.893	<0.001	1.620	2.269	
Injury Severity								
(Constant)	1.361	0.169		8.032	<0.001	1.020	1.702	0.105
JCD	0.422	0.183	0.325	2.303	0.026	0.053	0.792	

**Table 5:** Age and anthropometric outcomes in athletes ≤2 injuries and athletes with >2 injuries.

Variable	≤2 Injuries (*n* = 41)	>2 Injuries (*n* = 9)	*p*	ŋ^2^*p*
Age (years)	26.3 ± 7.5	26.7 ± 4.8	0.917	0.000
Height (m)	1.73 ± 0.09	1.79 ± 0.10	0.065	0.069
Body mass (kg)	63.1 ± 10.4	65.4 ± 12.1	0.551	0.007
BMI	21.0 ± 2.6	20.3 ± 0.7	0.446	0.012
Upper body length (cm)	83.4 ± 4.5	83.7 ± 8.2	0.908	0.000
Lower body length (cm)	89.5 ± 5.2	95.4 ± 4.1	0.002	0.179
Joint clearance discrepancies (cm)	0.80 ± 0.32	1.22 ± 0.53	0.003	0.172

Results are indicated as mean and standard deviation. All analysis were adjusted by race. *p*: Differences using one factor univariant ANOVA tests. BMI: Body mass index.

**Table 6:** Age and anthropometric outcomes in athletes with slight injury and serious injury.

Variable	Slight Injury (*n* = 16)	Serious Injury (*n* = 34)	*p*	ŋ^2^*p*
Age (years)	30.2 ± 8.7	25.4 ± 6.1	0.040	0.091
Height (m)	1.70 ± 0.08	175 ± 0.09	0.116	0.054
Body mass (kg)	59.0 ± 8.0	64.9 ± 10.9	0.085	0.064
BMI	20.3 ± 20.0	21.2 ± 2.7	0.337	0.021
Upper body length (cm)	82.1 ± 4.9	83.5 ± 5.2	0.404	0.16
Lower body length (cm)	88.1 ± 3.8	91.4 ± 6.0	0.074	0.069
Joint clearance discrepancies (cm)	0.68 ± 0.33	0.93 ± 0.33	0.026	0.105

Results are indicated as mean and standard deviation. All analysis were adjusted by race. *p*: Differences using one factor univariant ANOVA tests.

## Data Availability

Data sharing is not applicable to this article.

## Supplementary Materials

The following are available online at https://www.mdpi.com/2077-0383/10/5/1050/s1, Table S1: US national championships (minimum of “B” grade). Minimum score necessary. Table S2: US Junior Nationals. Minimum score necessary (U20).
