# Supplementary material for: The Role of Hip Joint Clearance Discrepancy as Other Clinical Predictor of Reinjury and Injury Severity in Hamstring Tears in Elite Athletes
Source: J Clin Med. 2021 Mar 4;10(5):1050. doi: 10.3390/jcm10051050 (PMC7961931; doi:10.3390/jcm10051050)
Supplement: Supplementary file 1 [file jcm-10-01050-s001.pdf]

Journal of
Clinical Medicine

MDPI

Supplementary Materials:

Table S1. US national championships (minimum of "B" grade). Minimum score necessary.

Event type	Minimum score necessary	
	**Men**	**Woman**
	"B"	
**100m**	10.36	11.48
**200m**	20.85	23.40
**400m**	46.25	52.70
**800m**	1:48.45	2:05.50
**1500m**	3:42.10	4:16.50
Mile	*3:59.50*	*4:37.00*
**5000m**	13:52.00	15:56.00
**10,000m**	29:01.30	34:00.00
**110m Hurdles**	13.89	13.25
**400m Hurdles**	50.75	57.95
**3000m Steeplechase**	8:45.70	10:18.00

Table S2. US Junior Nationals. Minimum score necessary (U20).

Event type	Minimum score necessary	
	**Men**	**Woman**
	"U20"	
**100m**	10.74	12.04
**200m**	21.64	24.64
**400m**	48.04	55.24
**800m**	1:51.00	2:09.00
**1500m**	3:48.00	4:28.00
Mile		
**5000m**	14:15.00	9:35.00
**10,000m**	30:30.00	16:30.00
**110m Hurdles**	14.74	14.24
**400m Hurdles**	53.34	60.44
**3000m Steeplechase**	9:10.00	11:00.00
